# Meristem identity and phyllotaxis in inflorescence development

**DOI:** 10.3389/fpls.2014.00508

**Published:** 2014-10-14

**Authors:** Madelaine E. Bartlett, Beth Thompson

**Affiliations:** ^1^Biology Department, University of Massachusetts AmherstAmherst, MA, USA; ^2^Biology Department, East Carolina UniversityGreenville, NC, USA

**Keywords:** inflorescence, phyllotaxis, inflorescence morphology, auxin, meristem identity, inflorescence evolution

## Abstract

Inflorescence morphology is incredibly diverse. This diversity of form has been a fruitful source of inquiry for plant morphologists for more than a century. Work in the grasses (Poaceae), the tomato family (Solanaceae), and *Arabidopsis thaliana* (Brassicaceae) has led to a richer understanding of the molecular genetics underlying this diversity. The character of individual meristems, a combination of the number (determinacy) and nature (identity) of the products a meristem produces, is key in the development of plant form. A framework that describes inflorescence development in terms of shifting meristem identities has emerged and garnered empirical support in a number of model systems. We discuss this framework and highlight one important aspect of meristem identity that is often considered in isolation, phyllotaxis. Phyllotaxis refers to the arrangement of lateral organs around a central axis. The development and evolution of phyllotaxis in the inflorescence remains underexplored, but recent work analyzing early inflorescence development in the grasses identified an evolutionary shift in primary branch phyllotaxis in the Pooideae. We discuss the evidence for an intimate connection between meristem identity and phyllotaxis in both the inflorescence and vegetative shoot, and touch on what is known about the establishment of phyllotactic patterns in the meristem. Localized auxin maxima are instrumental in determining the position of lateral primordia. Upstream factors that regulate the position of these maxima remain unclear, and how phyllotactic patterns change over the course of a plant's lifetime and evolutionary time, is largely unknown. A more complete understanding of the molecular underpinnings of phyllotaxis and architectural diversity in inflorescences will require capitalizing on the extensive resources available in existing genetic systems, and developing new model systems that more fully represent the diversity of plant morphology.

## Introduction

Inflorescence morphology is an important determinant of yield in agricultural settings, and fitness in natural ones (Wyatt, 1982; Harder et al., 2004). Inflorescence form shows a startling degree of diversity. This diversity is evident at both broad scales, such as across angiosperms, and at finer scales, such as in the grass family (Weberling, 1989; Vegetti and Anton, 2000).

Although all grass inflorescences are termed “panicles,” this descriptor belies the immense diversity within the family (Box 1). A recent paper from Kellogg et al. highlights the evolution of one aspect of inflorescence morphology in the grasses: the evolution of primary branch **phyllotaxis** (Kellogg et al., 2013). Through investigations of early inflorescence development in grass relatives, the authors establish that the ancestral primary branch phyllotaxis in the grass inflorescence was likely spiral. Most extant grasses still exhibit spiral phyllotaxis of primary branches, but there has been an evolutionary shift in the Pooideae. The earliest-diverging member of the Pooideae, *Brachyelytrum*, shows spiral phyllotaxis of its primary inflorescence branches. Following the divergence of *Brachyelytrum*, there is a shift to two-ranked phyllotaxis, with an angle of divergence less than 180°, and then a final evolutionary transition to true distichy (Box 1B) in the later diverging Pooids (including *Hordeum*, *Avena*, and *Brachypodium*). This paper highlights two phyllotactic shifts, one at the evolutionary level (spiral to distichous) and another at the developmental level (from vegetative to reproductive development).

Box 1 **A terminology primer.**

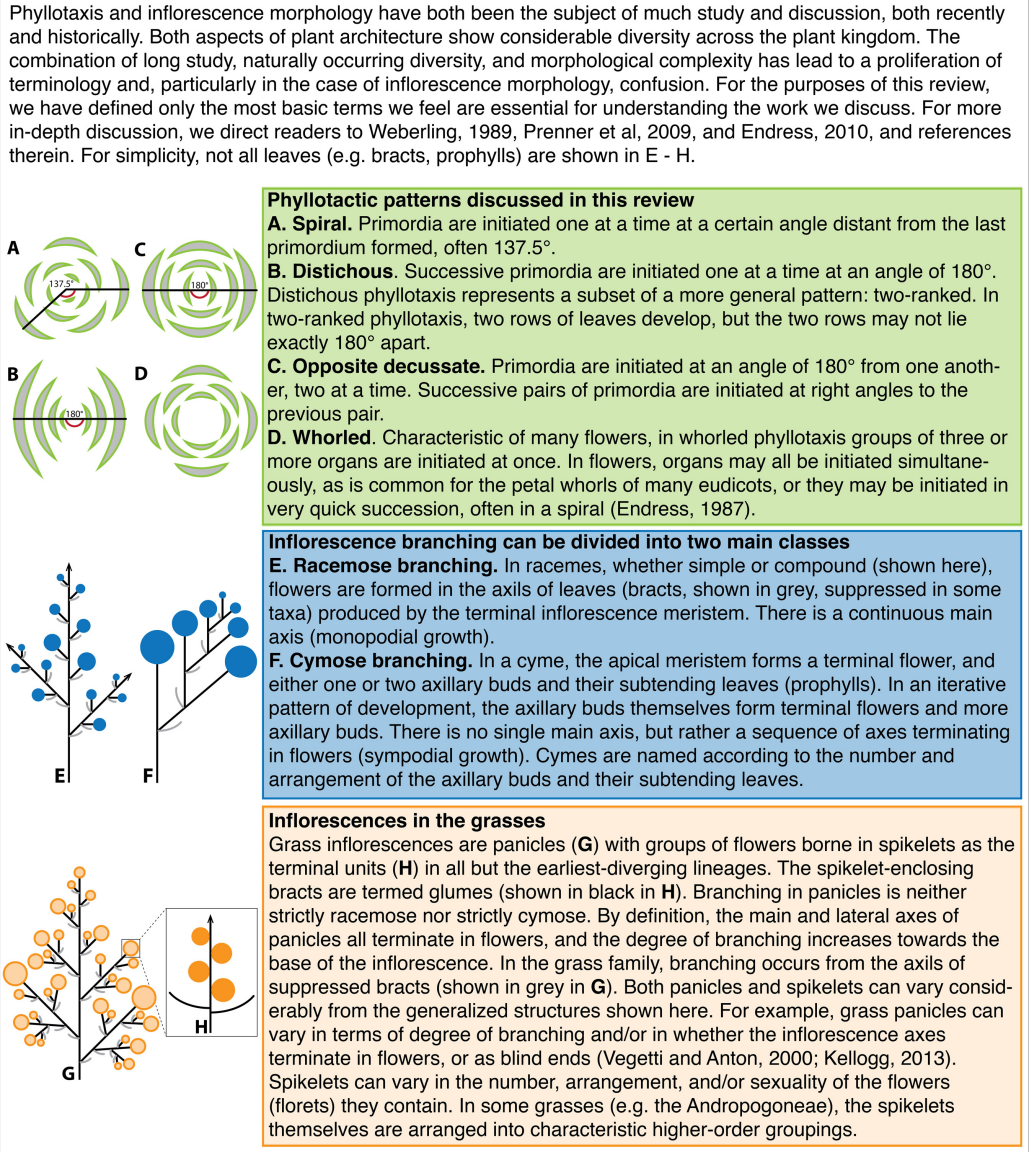

KEY CONCEPT 1 **Phyllotaxis**

The geometric arrangement of organs around a central axis. Phyllotactic patterns are determined largely by auxin and cytokinin gradients in the meristem. Altered hormone activity likely underlies developmental shifts in phyllotaxis.

The development of inflorescence morphology involves a complex interplay between a number of interacting processes: establishment of phyllotaxis and meristem identity, as well as variable patterns of cell division and expansion. As in all plant development, meristems and meristematic cells are of key importance in inflorescence development. Meristems are pools of undifferentiated cells that generate both more meristematic cells, as well as differentiated cells that form lateral organs. The particular character of a meristem can be described using the interrelated concepts of meristem identity and determinacy. **Meristem identity** refers to the type of primordia produced, whereas **meristem determinacy** refers to the number of primordia produced and whether or not the meristem is consumed in the production of primordia. For example, a meristem has floral meristem identity if it initiates floral organs and floral meristems are determinate because they are consumed in the production of a limited number of organ primordia. Each one of these factors can vary across both developmental and evolutionary time, leading to adult **inflorescence architectures** that show vast morphological diversity.

KEY CONCEPT 2 **Meristem identity and meristem determinacy**

Meristem identity refers to the type of primordia produced by a meristem, whereas meristem determinacy refers to the number of primordia produced and whether or not the meristem is consumed in the production of primordia.

KEY CONCEPT 3 **Inflorescence architecture**

The three dimensional arrangement of branches, leaves, flowers, and floral buds in an inflorescence (Box 1). Inflorescence architecture is determined in large part by shifting meristem identities over developmental time.

As highlighted by the Kellogg et al. (2013) paper, there is a need for understanding inflorescence morphology in terms of development, evolution, and at the intersection of these fields. There are a few examples where the adaptive value of individual aspects of inflorescence morphology have been investigated, but a very small number of systematic investigations into evolutionary patterns. This work was reviewed recently (Harder and Prusinkiewicz, 2013), and we will not revisit that topic here. Computational modeling and molecular genetic studies of inflorescence development in the grasses, petunia, tomato, and *Arabidopsis*, has lead to greater understanding of the regulatory logic that underlies inflorescence development (Rijpkema et al., 2006; Prusinkiewicz et al., 2007; Castel et al., 2010; Pautler et al., 2013; Tanaka et al., 2013; Zhang and Yuan, 2014). Similarly, strides have been made into understanding the molecular underpinnings of phyllotaxis, particularly in *Arabidopsis* (Kuhlemeier, 2007; Sassi and Vernoux, 2013; Traas, 2013). What has been missing is integration between these two closely allied topics.

Here, we attempt to seat what we know about the molecular and genetic factors that control phyllotaxis within the reigning paradigm of how we understand inflorescence development. We discuss recent investigations of inflorescence development in terms of shifting meristem identities, establishment of phyllotaxis and shifting phyllotactic patterns over the lifetime of a plant, and the close link between meristem identity and phyllotaxis.

## Shifting meristem identities during inflorescence development shape inflorescence architecture

Meristem identity changes during plant development. One key change in meristem identity is at the transition to flowering, where the vegetative meristem (VM) or shoot apical meristem (SAM) transitions to an inflorescence meristem (IM). A conceptual framework is emerging for understanding inflorescence development after this point in terms of further transitions between meristem identities. Computational modeling and molecular analyses of inflorescence development in the grasses, tomato (*Solanum lycopersicum*), petunia (*Petunia hybrida*), *Arabidopsis thaliana*, and *Pisum sativum* (Fabaceae) have revealed a framework of progressive changes in meristem identity underlying morphology.

An early model (Kellogg, 2000) portrayed inflorescence development as an iterative series of developmental switches that controlled the transition between meristem identities. In later computational modeling experiments, Prusinkiewicz et al. (2007) proposed a model wherein competency to flower is specified by a particular meristem's degree of *vegetativeness* (*veg*). For a meristem to transition to floral meristem fate, *veg* must drop below a threshold. *veg* in individual meristems could be specified by two major regulators of inflorescence development in *Arabidopsis*: *LEAFY* (*LFY*) and *TERMINAL FLOWER1* (*TFL1*). *LFY* and *TFL1* are transcription factors with roughly opposite roles. *LFY* promotes the transition from inflorescence to determinate floral meristem fate, while *TFL1* acts to repress floral meristem fate, effectively maintaining indeterminacy of the IM (Weigel et al., 1992; Bradley et al., 1997; Ratcliffe et al., 1999; Moyroud et al., 2010). In their model, *LFY* decreases *veg*, while *TFL* activity increases *veg*. Thus, variation in *veg*, a measure of meristem identity, acts over developmental time and morphological space to produce final inflorescence architecture (Prusinkiewicz et al., 2007). Common to both models (Kellogg, 2000; Prusinkiewicz et al., 2007) is the concept that variable meristem identity across the inflorescence is key in generating final structure. This concept continues to garner empirical support from work conducted in the grasses, the tomato family, and *Pisum*.

**Figure 1:**
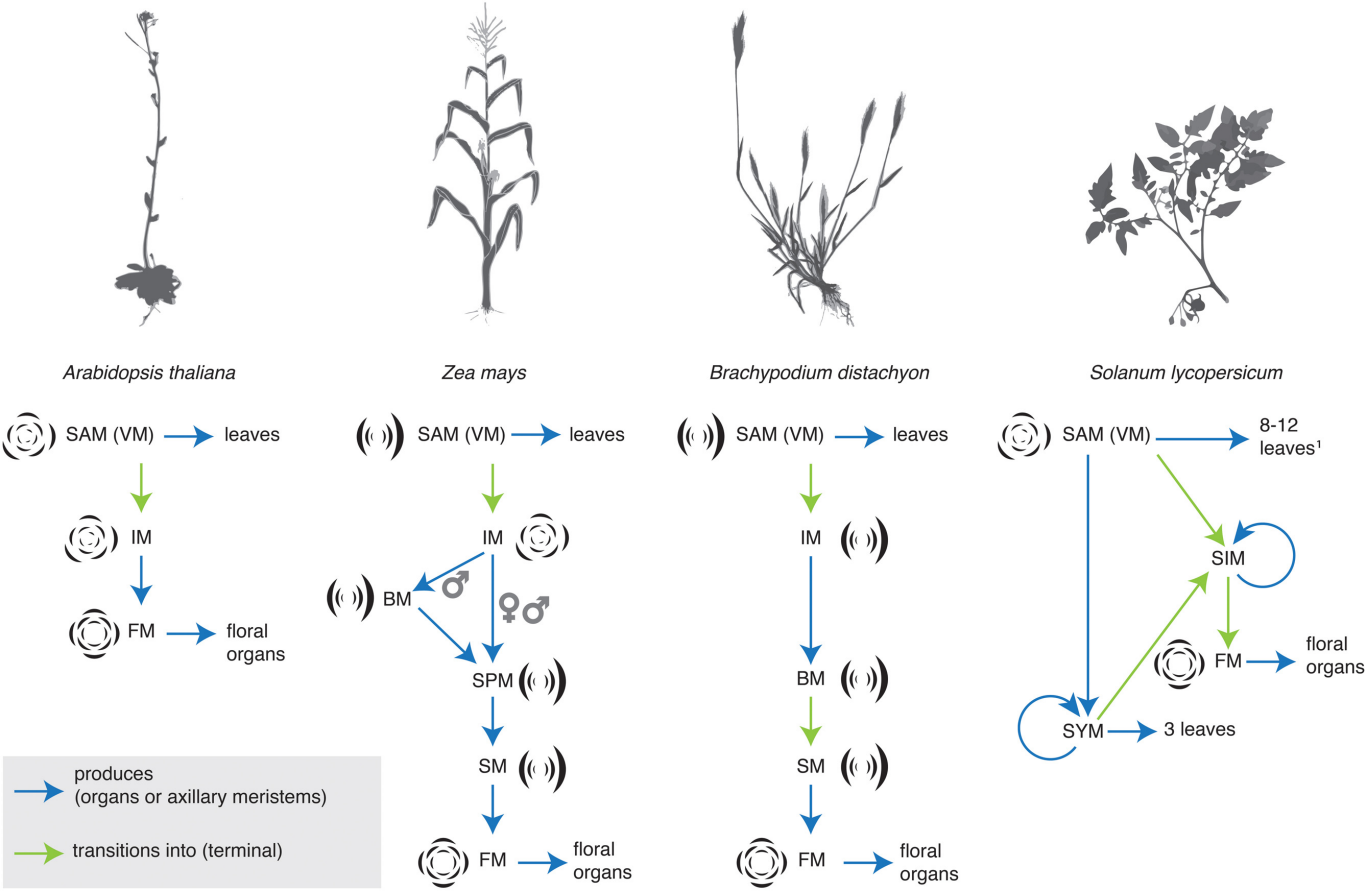
**Sterotypical shifts in inflorescence identity in *Arabidopsis thaliana*, maize, the Pooid grass *Brachypodium distachyon*, and tomato (*Solanum lycopersicum*)**. BM, branch meristem; FM, floral meristem; IM, inflorescence meristem; SAM (VM), shoot apical meristem (vegetative meristem); SIM, sympodial inflorescence meristem; SM, spikelet meristem; SPM, spikelet pair meristem; SYM, sympodial shoot meristem (Pnueli et al., 1998).

In the grasses maize and rice, studies of molecular genetics have revealed a regimented and precise transition between meristem identities during inflorescence development (Tanaka et al., 2013; Thompson, 2014). The grasses bear their flowers in spikelets (Box 1). Grass inflorescences undergo a series of meristem transitions, generalized as branch (BM), to spikelet (SM), to floral meristem (FM) identity, although there are variations between taxa and male and female inflorescences. Maize, and its allied grasses in the Andropogoneae, bear their spikelets in pairs, and thus possess a separate spikelet pair meristem (SPM) identity (Figure 1). Over the years, many key developmental regulators that control these transitions have been identified. The se developmental regulators have been described and cataloged in a number of recent reviews, and so we do not discuss them here at length (reviewed in Pautler et al., 2013; Tanaka et al., 2013; Teo et al., 2014; Thompson, 2014). Meristem identity in the inflorescence is controlled by a sequentially active series of genes. Some genes and gene functions are conserved between maize and rice, while others appear unique to a subset of grasses. For example, both *BRANCHED SILKLESS1* (*BD1*) and its rice ortholog, *FRIZZY PANICLE1* (*FZP1*) regulate SM identity and determinacy. In *bd1* and *fzp1* mutants, SMs take on a more BM-like identity (Chuck et al., 2002; Komatsu et al., 2003). In contrast, the *RAMOSA* (*RA*) genes are key regulators of SPM (*RA1, RA2, RA3*) and SM (*RA3*) determinacy (Vollbrecht et al., 2005; Bortiri et al., 2006; Satoh-Nagasawa et al., 2006). Rice has no ortholog of *RA1*, indeed *RA1* appears to be unique to the Andropogoneae (Vollbrecht et al., 2005). The homologs of *RA2* and *RA3* have not been characterized in rice, but the barley *RA2* ortholog, *Vrs4*, appears to play an analogous role in regulating meristem determinacy in the inflorescence (Koppolu et al., 2013).

In the Solanaceae (tomato, peppers, and petunia), several genetic mutants that disrupt inflorescence architecture have been characterized and cloned (reviewed most recently in Cohen et al., 2012; Park et al., 2014). Transcriptional evidence also supports the concept of shifting meristem identity in tomato inflorescences (Park et al., 2012). The branching pattern in tomato is cymose (but see Prenner et al., 2009) and the progression from inflorescence to floral meristem occurs iteratively across the inflorescence through a stereotypical progression of meristem identities (Figure 1). Transcriptional dynamics across this progression reveal that these meristems are distinct both in their products and in their molecular profiles. Variation in meristem identity across the inflorescence, expressed as differing maturation states, may be key in generating final inflorescence architecture, both in *Solanum* and in the broader Solanaceae (Park et al., 2012, 2014). One important point arising from this work (Park et al., 2012), and transcriptional profiling of *ra* mutants in maize (Eveland et al., 2014) is that the molecular sequence of meristem identities is much more fine-grained than the morphologically identifiable sequence of identities. Morphologically distinct meristem identities serve as useful markers, but they overlie a much larger degree of change that occurs at the molecular level.

The first IM identity gene was recently cloned in the legume *P. sativum*. *Pisum*, and indeed most legumes, bears its flowers in compound racemes (Box 1E). The IM produces secondary inflorescence meristems that ultimately produce flowers. *vegetative1* (*veg1*) plays a role in specifying secondary inflorescence meristem identity. In v*eg1* mutants, VMs form in the position of secondary inflorescence meristems, which never produce flowers. *VEG1* encodes an AGL79-like MADS-box gene that appears to be eudicot-specific (Berbel et al., 2012).

Apart from meristem identity, positional effects within an inflorescence have long been known to play a role in determining architecture. By studying inflorescence morphology in thousands of species, Troll inferred a generalized inflorescence architecture composed of four zones (the innovation, inhibition, enrichment, and terminal zones), occupying distinct regions of the inflorescence (Troll, 1969; Weberling, 1989). The concept of distinct inflorescence zones is supported by studies of both genetic mutants, and natural diversity. For example, in *ra1* mutants, later meristems don't have the capacity to respond to the lack of *ra1* in the same way as earlier ones do, leading to a grade of branching in the inflorescence (Vollbrecht et al., 2005). In the Solanaceae, inflorescence position also plays a key role in determining floral meristem fate and floral sexuality (Diggle and Miller, 2004). The molecular nature of these positional signals remains unknown (transcriptional profiling experiments are giving hints, see Eveland et al., 2014). What seems clear, however, is that positional information along the proximo-distal inflorescence axis interacts with meristem identity networks to generate inflorescence architecture.

Modulation of meristem identity allows for morphological differentiation in the inflorescence (Kellogg, 2007). A striking example of this can be seen in plants that produce vegetative plantlets termed “bulbils” in place of flowers in their inflorescences (Elmqvist and Cox, 1996). In *Titanotrichum oldhamii* (Gesneriaceae), for example, later “floral” meristems are replaced with bulbils under short day conditions. A *LFY* homolog is downregulated in these bulbils, as expected for a transition to VM identity (Wang et al., 2004). Taxa where bulbils form often live in alpine, arctic, or arid habitats. Thus, this innovation in inflorescence architecture might represent an adaptation to a short growing season, or to the heterogeneity characteristic of these environments (Elmqvist and Cox, 1996). A more familiar example of the reversion of a reproductive meristem to a vegetative meristematic state can be found in pineapples (*Ananas comosus*, Bromeliaceae), where vegetative growth continues after flowering (Bell and Bryan, 1991).

These examples illustrate the lability of meristem identity under certain conditions. Altering and repurposing existing meristem identity networks is one pathway evolution can take within a clade (e.g., bulbils, inflorescence diversity in the Solanaceae) (Park et al., 2014). Between clades, unique meristem identities seem to be specified by a mix of both unique and conserved genes. Some of the same genes were identified in transcriptional profiling of both *ra* mutants in maize, and in tomato inflorescences (e.g., TCP transcription factors and *ga-2 oxidase*) (Park et al., 2012; Eveland et al., 2014). However, a number of the upstream regulators seem unique to specific clades (e.g., *ra1* and the SPM in maize, *veg1* and the secondary inflorescence meristem in *Pisum*). As researchers sample further there may be more examples of genetic conservation, but we need more genetic models, throughout the angiosperms, to find the genes. For example, thyrses (cymose branching on a racemose main axis, Endress, 2010) are fairly common, yet remain unmentioned in the developmental genetic literature.

## Phyllotaxis is one aspect of meristem identity

Meristem identity and phyllotaxis are intimately linked. This link has been revealed by studies of both natural diversity and genetic mutants. In *Arabidopsis*, the grasses, and *Solanum*, the meristem identity transitions that determine inflorescence architecture are often accompanied by changes in phyllotaxis (Figure 1). In the grasses, for example, the VM initiates leaves in a distichous phyllotaxis, IMs produce branches spirally (except in the Pooideae, see above Kellogg et al., 2013), branch meristems produce either branches or spikelets in two ranks, and floral organs are patterned in a whorled phyllotaxis (although they may be initiated spirally, see Box 1). The main axis eventually switches to producing spikelets in two ranks (Box 1), except in the maize tassel, where the main axis continues to produce spikelet pairs spirally (Kellogg et al., 2013).

One of the phyllotactic shifts observed in grasses is common to many flowering plants. The transition from IM identity (indeterminate, initiates floral meristems) to floral meristem identity (determinate, initiates floral organs) is often accompanied by a shift to whorled phyllotaxis (Figure 1) (Bell and Bryan, 1991; but see Endress, 1987; Specht and Bartlett, 2009). Earlier in development, dicotyledonous plants often undergo a shift in phyllotaxis from opposite decussate to spiral phyllotaxis because cotyledons and the first leaves are initiated as opposite pairs (Bell and Bryan, 1991). Furthermore, mutants or double mutants that significantly perturb meristem fate often cause a dramatic shift in phyllotaxis. For example, in *Arabidopsis lfy* mutants and in *Antirrhinum floricaula (flo)* and *squamosa (squ)* mutants, floral meristems are converted to IMs, and whorled floral phyllotaxis is replaced by spiral phyllotaxis (Coen et al., 1990; Huijser et al., 1992; Weigel et al., 1992; Carpenter et al., 1995). Similarly, the maize *bde zag1* and *ifa1 ids* double mutants cause a determinate meristem (FM or SM) to revert to an indeterminate branch-like meristem, and have BM-like phyllotaxis, rather than FM-like whorled phyllotaxis (Laudencia-Chingcuanco and Hake, 2002; Thompson et al., 2009).

These meristem identity and phyllotactic shifts must be tied into the regulatory pathways that regulate the transition from juvenile to adult growth, and in turn from vegetative to reproductive growth. The juvenile to adult transition in plants is largely controlled by the activity of two opposing microRNAs (miRNAs) (Wu and Poethig, 2006; Chuck et al., 2007; Wang et al., 2011; Zhang et al., 2011; Fu et al., 2012; Shikata et al., 2012; Xie et al., 2012; Poethig, 2013). MiR156 promotes juvenile development, while miR172 promotes reproductive development. Mutants that perturb this network also perturb phyllotaxis. For example, the dominant maize mutant *Corngrass1 (Cg1)* overexpresses miR156 and extends the juvenile phase. *Cg1* inflorescences, while still spiral, have abnormal phyllotaxis and do not produce ordered rows of meristems (Chuck et al., 2007). These phyllotactic transitions governed by phase transitions can also be observed by studying natural diversity. In *Eucalyptus globulus*, for example, the transition from juvenile to adult growth is accompanied by a shift from opposite decussate to spiral phyllotaxis (Zotz et al., 2011).

Meristem determinacy mutants also affect phyllotaxis. Mutants that increase meristem indeterminacy due to increased stem cell activity often have abnormal phyllotaxis (e.g., *unusual floral organs (ufo)* in *Arabidopsis, bearded-ear1 (bde)* in maize, and *OsMads3/53* in rice), albeit these mutants usually do not exhibit a discrete shift from one phyllotactic pattern to another (Levin and Meyerowitz, 1995; Thompson et al., 2009; Dreni et al., 2011). Meristem determinacy is impacted not only by stem cell activity, but also by cell partitioning to the three components of the phytomer (leaf/bract/prophyll, axillary meristem, and stem). The links between cell partitioning, determinacy and phyllotaxis are illustrated by analysis of the maize *tasselsheath4 (tsh4)* mutant. *tsh4* suppresses bract outgrowth in the inflorescence and in *tsh4* mutants, cells that would normally be partitioned to the axillary meristem are partitioned to the bract. This reallocation of cells results in increased meristem determinacy (fewer lateral primordia are produced), as well as disruption of phyllotaxis in the ear (Chuck et al., 2010). Intriguingly, *tsh4* is targeted by miR156, which as mentioned above plays a fundamental role in the juvenile to adult transition. *tsh4* also regulates the meristem determinacy gene *ra2*. In *tsh4* mutants, expression of RA2 protein is expanded outside of its normal domain in the SPM, into the de-repressed bract (Chuck and Bortiri, 2010).

Together, these analyses highlight **the integrated nature of plant development**. Developmental programs that regulate meristem determinacy and phyllotaxis cannot be considered independently of meristem identity. Determinacy and phyllotactic pattern are not imposed on a particular meristem, but are rather intrinsic to a particular meristem. In addition, these examples illustrate that the programs that regulate life span progression must also be tied into phyllotactic programs. Recently, great progress has been made in understanding the molecular underpinnings of these phyllotactic patterns.

KEY CONCEPT 4 **The integrated nature of meristem development**

Meristem identity, meristem determinacy, and phyllotaxis are intimately linked. Regulation of meristem fate and phyllotaxis appear to be linked into the developmental programs that control life progression.

## Auxin and molecular models of phyllotaxis

The robust and reproducible nature of phyllotaxis has intrigued and fascinated biologists, mathematicians, and physicists for centuries (Adler et al., 1997). Numerous models have been proposed to describe phyllotaxis. Central to these models is the idea that emerging primordia create an inhibitory field that suppresses the formation of new primordia (Hofmeister, 1868; Snow and Snow, 1962; Douady and Couder, 1996; Adler et al., 1997). The position of the next primordium is therefore determined by where the inhibitory field is lowest. This concept has been confirmed by microsurgery and laser ablation experiments that disrupt primordia and also phyllotaxis (Snow and Snow, 1932; Reinhardt et al., 2005).

Work conducted in the past decade has greatly increased our understanding of the molecular mechanisms that regulate primordium initiation and phyllotaxis. The phytohormone auxin has emerged as a central player in the regulation of both processes (for detailed reviews see Kuhlemeier, 2007; Sassi and Vernoux, 2013; Traas, 2013). Auxin is required to initiate all lateral primordia. Mutants that disrupt auxin synthesis or transport have reduced numbers or completely lack lateral primordia in *Arabidopsis*, petunia, and maize (Okada et al., 1991; Galweiler et al., 1998; Tobeña-Santamaria et al., 2002; Friml et al., 2004; Cheng et al., 2006; McSteen et al., 2007; Gallavotti et al., 2008a; Phillips et al., 2011). At least in some contexts, primordium initiation defects can be rescued by application of exogenous auxin, indicating that auxin plays an instructive role in the initiation of lateral primordia (Reinhardt et al., 2000). Indeed, lateral primordia initiate at regions of high auxin, and high auxin activity (as measured by the synthetic auxin reporter, *DR5*) coincides with primordium initiation (Heisler et al., 2005; Gallavotti et al., 2008b; O'Connor et al., 2014).

The mechanisms by which auxin maxima are formed are complex and include both auxin synthesis and auxin transport. Central players in auxin localization are the PIN-FORMED (PIN) auxin efflux carriers, of which PIN1 appears to be most critical for initiation of lateral primordia during reproductive development (Okada et al., 1991; Galweiler et al., 1998; Reinhardt et al., 2000; Vernoux et al., 2000). PIN1 is asymmetrically localized in the cell in response to auxin. During the initiation of lateral primordia, PIN1 forms convergence points that are instrumental in creating auxin maxima. In this developmental context, PIN1 is asymmetrically localized on cell membranes, and is proposed to pump auxin up a concentration gradient to ultimately form auxin maxima (Heisler et al., 2005; O'Connor et al., 2014). In addition to determining the position of primordia, these auxin maxima function as auxin sinks and deplete the surrounding cells of auxin. Polarized auxin transport thus provides mechanistic support for the “inhibitory field” hypothesis, and explains why two primordia cannot form adjacent to one another (Reinhardt et al., 2003; Reinhardt, 2005; De Reuille et al., 2006).

Auxin transport and regulation of phyllotaxis is undoubtedly more complex, as evidenced by the existence of multiple PIN1-like proteins in multiple contexts (Bennett et al., 2014a). In Arabidopsis, PIN1 is the main PIN that functions in the SAM (Benková et al., 2003; Reinhardt et al., 2003; Scarpella et al., 2006; Guenot et al., 2012). Outside of the Brassicaceae, Sister of PIN1 (SoPIN1, absent from the Brassicaceae) seems to have a more important role in auxin distribution on the meristem surface (O'Connor et al., 2014). In the grasses, PIN1 has been duplicated, resulting in three PIN proteins that regulate auxin distribution in developing inflorescences. In *Brachypodium* and maize, SoPIN1 is localized to the surface, while PIN1a and PIN1b are more prominent in internal tissues and likely function in vasculature formation. A model that successfully recapitulates experimental evidence varies how each PIN transporter responds to auxin (O'Connor et al., 2014). For example, SoPIN1 moves auxin up the gradient and PIN1a and PIN1b canalize auxin flow by transporting auxin in the direction of greatest auxin flux. Thus, the number, localization, and activity of PIN proteins are likely important drivers in the positioning of auxin maxima, and perhaps in the evolution of different phyllotactic patterns.

Changes in PIN localization and auxin signaling pathways also have the potential to alter auxin activity and phyllotaxis during development. Very little is known about the molecular mechanisms by which PIN proteins are localized in response to auxin (Michniewicz et al., 2007; Robert et al., 2010), which is a major limitation in generating biologically meaningful auxin transport models (Bennett et al., 2014b). Furthermore, not all cells are competent to respond to auxin, and localization of auxin signaling molecules might also play a role in phyllotaxis. For example, cells within the central zone have high auxin levels, but do not respond to auxin and activate auxin reporter genes (De Reuille et al., 2006; Brunoud et al., 2012).

Auxin synthesis also plays an important role in the initiation of lateral primordia. In maize, *vanishing tassel2* and *sparse inflorescence1* encode auxin biosynthesis enzymes and both *vt2* and *spi1* mutant inflorescences have dramatically reduced numbers of lateral primordia (Gallavotti et al., 2008a; Phillips et al., 2011). Interestingly, although both *vt2* and *spi1* function in auxin biosynthesis, they are not co-expressed in the same domain. *vt2* is expressed in the epidermal layer of initiating primordia, prior to any morphological outgrowth, whereas *spi1* is expressed throughout incipient SPMs, and has restricted expression in the SM and FM. Thus, *vt2* and *spi1* appear to make unique contributions to auxin levels and primordium initiation in developing inflorescences.

Players upstream and downstream of auxin are starting to be identified. The PLETHORA (PLT) transcription factors are required for spiral phyllotaxis in the *Arabidopis* shoot and are required to upregulate two auxin biosynthesis genes (*YUC1* and *YUC4)* in the central zone of the SAM (Pinon et al., 2013). As mentioned above, *Arabidopsis* goes through a phyllotactic shift common to many dicotyledonous plants. The cotyledons and first two leaves of *Arabidopsis* are initiated in an opposite decussate phyllotaxis and all subsequent leaves are initiated spirally. *plt3plt5plt7* triple mutants delay this phyllotactic shift (Prasad et al., 2011). Most models of phyllotaxis only take into account auxin transport (De Reuille et al., 2006; Jönsson et al., 2006; Smith et al., 2006), but analysis of auxin biosynthesis mutants such as *plt* and *yuc*, indicate that auxin biosynthesis likely plays a significant role (Cheng et al., 2006; Pinon et al., 2013). Along this line, the INDETERMINATE DOMAIN (IDD) transcription factors IDD14, IDD15, and IDD16 have recently been implicated as directly regulating both *PIN1* and *YUC5* in the combinatorial control of auxin signaling and organ growth in *Arabidopsis* (Cui et al., 2013).

Auxin promotes organogenesis at least in part by affecting cell wall rigidity. Demethylesterification of pectin, which increases elasticity in the meristem, is necessary and sufficient for primordium initiation. Overexpression of pectin methylesterase 5 results in increased numbers of lateral primordia, with aberrant phyllotaxis (Peaucelle et al., 2008, 2011). Auxin promotes organ outgrowth at least in part by promoting pectin demethylesterification, since PME13 overexpression lines (which block demethylestermifcation) have pin-like inflorescences that fail to form primordia in response to exogenous auxin (Braybrook and Peaucelle, 2013). PIN1 also seems to respond to mechanical cues in the meristem. PIN1 localization is correlated with the direction of microtubule arrays, and genetic or chemical disruption of the cell wall disrupts PIN1 localization (Heisler et al., 2010; Braybrook and Peaucelle, 2013). Thus, differences in cell wall composition and mechanical forces within the meristem may also play an instructive role in creating auxin maxima and determining phyllotaxis. Mechanical forces have a demonstrated effect on the appearance of phyllotactic pattern post-initiation. Lateral primordia may be initiated in one phyllotactic pattern, but twisting of the stem after initiation can lead to an altered final leaf position (Bell and Bryan, 1991; Landrein et al., 2013).

In addition to auxin, the phytohormone cytokinin plays a critical role in the regulation of meristem size and phyllotaxis. The maize *aberrant phyllotaxy1 (abph1)* and the rice *decussate (dec)* mutants stably change leaf phyllotaxis from distichous (alternate) to opposite decussate (Jackson and Hake, 1999; Itoh et al., 2012). Both *abph1* and *dec* mutants are defective in cytokinin signaling. *abph1* encodes a cytokinin-inducible type A response regulator (ARR) that negatively regulates cytokinin signaling (Giulini et al., 2004). *dec* encodes a plant-specific protein that functions in cytokinin signaling, although the exact role of the DEC protein is unknown (Itoh et al., 2012). In addition to phyllotactic defects, both *abph1* and *dec* mutants have enlarged SAMs, lending support to the idea that meristem size impacts phyllotaxis (Snow and Snow, 1932). Indeed, *Arabidopsis* mutants with enlarged meristems often have perturbed or shifted phyllotaxes (Leyser and Furner, 1992; Goldshmidt et al., 2008; Mandel et al., 2014). Cytokinin also plays a role in meristem size and phyllotaxis in *Arabidopsis*. Cytokinin signaling has been directly linked the transcriptional regulatory network that controls stem cell number. The transcription factor WUSCHEL (WUL) controls the size of the stem cell niche by negatively regulating stem cell number and directly represses *ARR* genes (Leibfried et al., 2005). However, mutants that disrupt cytokinin signaling in *Arabidopsis* are not as severe as their grass counterparts and do not show a discrete phyllotactic shift (Leibfried et al., 2005; Zhao et al., 2010).

The cytokinin network intersects with the auxin network and this interplay seems to be particularly important in determining the timing between primordium initiation events, or the plastochron. Since phyllotaxis is determined by a combination of spatial determinates (where primordia arise on the meristem periphery) and timing determinates (time between subsequent primordium initiation events), this interplay is a key determinate of phyllotaxis. In maize *abph1* mutants, both PIN1 and auxin levels are decreased, suggesting that cytokinin negatively regulates PIN1 (Lee et al., 2009). This observation led the authors to hypothesize that the phyllotactic shift in *abph1* mutants may be due to a delay in organ initiation, not just due to an increase in meristem size. Similarly, reduced plastochron duration in the *corkscrew1* (*cks1*) mutant of maize occasionally leads to a shift to opposite decussate phyllotaxis (Alexander et al., 2005). In *Arabidopsis*, cytokinin does not appear to affect PIN1 localization or levels, but inhibitory cytokinin signaling fields are required downstream of auxin to determine the timing of primordium initiation (Besnard et al., 2014a) and auxin and cytokinin appear to function synergistically to initiate primordia (Besnard et al., 2014b). Direct links between auxin and cytokinin have been established in *Arabidopsis* shoots, in which the auxin response factor (ARF), ARF5/MP, directly modulates the expression of two *ARR* genes, *ARR7* and *ARR15* and the cytokinin inhibitor, *AHP6* (Zhao et al., 2010; Besnard et al., 2014a). The mechanism of crosstalk between auxin and cytokinin pathways has not yet been determined in the grasses.

Molecular analyses clearly indicate that the position of auxin maxima determines the position of lateral primordia and thereby phyllotactic pattern. Cytokinin also appears to play a key role in generating robust phyllotactic patterns. How then do phyllotactic patterns change, both over the course of development, and across evolutionary time? The positioning of auxin maxima, modulated by cytokinin signaling, appears to be key. This suggests that regulation of auxin synthesis and transport is different in meristems with different phyllotactic patterns. Differences in auxin transport and regulation are likely due to differential expression or regulation of PIN proteins or auxin biosynthesis genes (e.g., through the *PLT* or *IDD* genes), which can differ depending on meristem identity. Thus, phyllotaxis is intimately linked to and one aspect of meristem identity.

## Moving forward

Inflorescence architecture is a major determinant of reproductive success and fitness (Wyatt, 1982; Harder et al., 2004) that cannot be understood by looking only to *Arabidopsis*, or indeed only to the grasses. The development of new model systems has opened up possibilities for investigating meristem dynamics and inflorescence development in a range of taxa with divergent morphologies. Exploring this diversity will allow for a more nuanced understanding of the underlying principles governing shifting meristem identity in particular, and plant development in general.

One important aspect of meristem identity and plant architecture, both in the inflorescence and in the vegetative shoot, is phyllotaxis. Developmental and molecular biology have converged with mathematical modeling to generate a framework to describe the role of auxin in initiating primordia and the establishment of phyllotaxis. Very little is known, however, about how phyllotactic shifts occur either within species (over developmental time) or between species (over evolutionary time). One of the first steps to understanding evolutionary shifts in phyllotaxis is to continue to trace phyllotactic patterns across the tree (Kellogg et al., 2013), at both broad and fine scales.

How do phyllotactic shifts occur? We propose that phyllotaxis is intimately connected to meristem identity. Since phyllotaxis is ultimately determined by auxin (and perhaps cytokinin) signaling within the meristem, meristems with different identities (and phyllotaxes) must also differentially regulate auxin accumulation and signaling. Indeed, in ChIP-Seq and RNA-Seq experiments, transcriptional regulators of meristem identity (e.g., SEP4, RA1) directly regulate genes in the auxin regulatory network (Kaufmann et al., 2009; Eveland et al., 2014). KNOTTED1 (KN1), a canonical regulator of meristematic activity (Vollbrecht et al., 1991, 2000), binds and modulates many genes in hormonal pathways, particularly genes in the auxin pathway (Bolduc et al., 2012). In *ra* mutants, auxin signaling is perturbed before any morphological phenotype can be observed (Eveland et al., 2014). Only a small fraction of hormone-associated genes were regulated by RA1, but a significant fraction of the auxin-related genes were bound and co-regulated by RA1 and KN1, suggesting a link between auxin signaling and meristem determinacy (Bolduc et al., 2012; Eveland et al., 2014). Three *IDD* genes (mentioned above) are direct targets of RA1 (Eveland et al., 2014). Thus, the remodeling of auxin signaling networks (and therefore phyllotaxis) might be one aspect of how meristem identity is realized. Similarly, differences in auxin biosynthesis, transport, and signaling likely account for phyllotactic differences between species. To address these questions, we must integrate molecular and genomic approaches with a deep understanding of plant morphology and diversity, and expand analyses beyond a few model species. These endeavors will undoubtedly shed light onto how plants generate their amazing diversity of form.

### Conflict of interest statement

The authors declare that the research was conducted in the absence of any commercial or financial relationships that could be construed as a potential conflict of interest.
